# Understanding sociodemographic differences in climate behavior, climate policy acceptance, and political participation

**DOI:** 10.1016/j.joclim.2024.100353

**Published:** 2024-10-16

**Authors:** Lena Lehrer, Lennart Hellmann, Cornelia Betsch

**Affiliations:** a Health Communication, Implementation Research, Bernhard Nocht Institute for Tropical Medicine, Health Communication Working Group, Bernhard Nocht Straße 74, 20359 Hamburg, Germany; b Health Communication, Institute for Planetary Health Behaviour, University of Erfurt, Universität Erfurt, Nordhäuser Straße 63, 99089 Erfurt, Germany

**Keywords:** Target-group segmentation, Social marketing, Environmental psychology, Public-health messaging, Sociodemographic factors in climate-change communication, Climate-change communication strategies

## Abstract

**Introduction:**

To mitigate the global health threat posed by climate change, multifaceted responses are required. In previous work, older, male, and highly educated individuals were shown to exhibit greater general readiness to act against climate change; the same is true for people living in larger communities. Yet, the sociodemographic effects may vary for the single facets of the readiness to act against climate change—namely individual climate-friendly behavior, acceptance of climate-protection policies, and political participation.

**Methods:**

Data from four waves (2022–2023) of the Planetary Health Action Survey (PACE) were analyzed (*N* = 3,830, nonprobabilistic representative German sample). Sociodemographic variables (gender, age, community size, education and others) and the three abovementioned facets of the readiness to act against climate change were assessed to examine their relationships.

**Results:**

Patterns varied between the indicators. Women and older individuals exhibited more climate-friendly behavior and policy acceptance. In contrast, levels of political participation were higher for men and younger individuals. Higher education was linked to less sustainable behavior, greater policy acceptance, and higher participation, while municipality size was only linked to the latter two. Additional analyses explore the relations between age and the constructs at item level.

**Conclusions:**

Findings underscore the importance of a nuanced understanding of people's readiness to act against climate change. Depending on communication objectives, different target groups may be relevant, e.g., do campaigners want to increase political participation vs. change individual behaviors. While older individuals exhibit greater readiness to act via individual behavior and policy acceptance, it is important to include young people in climate-communication efforts.

## Highlights

• Different facets of climate action were linked to sociodemographics.
• Women and older participants showed more individual mitigation and policy approval.
• Male gender and lower age correlated with higher political participation.
• It is important to appeal to younger generations in climate-change communication.
• Target groups should be considered in conjunction with the targeted climate protection behavior.

## 1 Introduction

Climate change affects human health worldwide [[1], [2], [3]] and scientists agree immediate and drastic measures are needed to mitigate climate change. To achieve this goal, a multifaceted approach is essential. Individual behaviors, such as air travel, car usage, and dietary choices serve as direct levers of change [3]; however, while individual behaviors are vital, it is widely acknowledged they are insufficient to drive the societal transformation needed for sustainability. A focus on individual actions has been identified as a reason for the slow and ineffective response to climate change [[4], [5], [6]] and implementing effective political measures is crucial to alleviate climate change with public acceptance of and compliance with policies being key factors [7,8]. Furthermore, in democracies, citizens can engage in politics through institutional means, such as elections and petitions, or by participating in discussions and demonstrations. Therefore, in the present study, we seek to understand individual climate-friendly behavior, acceptance of climate-protection policies, and climate-specific political participation as indicators of the readiness to act against climate change [4,9,10].

Understanding which factors affect readiness to act regarding climate change [9] and its indicators as well as which sociodemographic factors are related to high or low values in these constructs [11] allows the targeting of climate communication and interventions. A segmentation and targeting approach has been suggested as a simple and effective way to make climate-change communication impactful [12] and the use of audience-specific (targeting) or individually customized (tailoring) communications has proven effective to address many behaviors in the context of climate change [13] and health [14,15]. Still, while some studies have categorized target groups based on attitudes toward climate change [16,17], large-scale communication campaigns require creating target groups based on readily observable characteristics, such as sociodemographic factors [11]. Understanding target groups is important to select optimal communication channels, visuals, style and tone of communications. In this study, our goal was to resolve uncertainties from prior research by focusing on socio-demographics. Earlier results identified that being young, male, having lower education, and living in smaller communities (less than 100,000 inhabitants) are related to lower general readiness to act against climate change [11], making these pertinent target groups for climate- and health-communication efforts. Moreover, research using the conceptualization of the readiness to act found that older individuals showed higher readiness to act than their younger counterparts. This contradicts previous findings in mainly German- and English-speaking samples [18] and visible movements such as Fridays for Future that may fuel the idea that it is mostly the young who engage in climate action [19]. As readiness to act is a broad concept encompassing individual behavior, policy acceptance and political participation, the question arises whether the sociodemographic effects are similar or different for single indicators of the readiness to act. Therefore, in this study we advance previous work by separating readiness to act into its components, treating them as single outcome variables and separately assessing the effects of sociodemographic characteristics on these indicators. We also focus on age to elucidate conflicting findings. In sum, our goal is to facilitate effective communication on climate change and health by identifying target groups based on specific patterns of sociodemographic variables.

## 2 Methods

### 2.1 Design

The Planetary Health Action Survey (PACE) monitors Germans’ readiness to act against climate change from a psychological perspective. It is designed as a repeated cross-sectional online survey with a nonprobabilistic sample of about *N* = 1000 for each wave, which is a quota representative of the German population for age × gender and federal state. At the time of writing, 19 waves have been conducted; only four are used here as previous ones were used to develop the instruments and varied slightly in the assessed items and constructs [9,10].

### 2.2 Measures

The supplement contains all measures used in this study, both in the original German and in the English translation. The items were randomized within blocks; all items had to be answered to proceed. Detailed information on the scales’ development can be found in the PACE study protocol [10].

*Sociodemographic characteristics*. Participants provided the following information: gender, age, federal state, education, number of inhabitants in their community, parental status, and migration history. Age was assessed continuously with an open text field and summarized into four groups (18–29 years, 30–49 years, 50–64 years, 65–74 years) for most analyses [20]. Education was categorized into three levels: low (up to 9 years of school education), medium (at least 10 years without a university entrance qualification), and high (at least 10 years with a university entrance qualification). Employment status, household net income per month, and presence of chronic disease were also assessed. The participants were allowed to skip the questions regarding migration, income, and chronic disease. For the analysis, adjusted household income was used (income divided by the square root of the household size [21]).

*Readiness to act*. Readiness to act consisted of individual behavior, acceptance of policy measures, and political participation [9].Variables were measured separately via several items (see below). Higher values indicate greater readiness to act.

*Individual behavior*. This revised version [22] of the short impact-based scale of environmental behavior [SIBS; 23] contains 21 questions relating to four different domains of climate impact (housing, mobility, nutrition, and other consumption). In the analysis, a weighted mean score was calculated for the domains and as an overall mean. The relative contributions to CO_2_ footprint were used as weights for single behaviors and categories in the total score. Thus, the mean score (*M* = 4.66, *SD* = 0.67) approximates the CO_2_ -footprint as assessed by a CO_2_-calculator [23].

*Policy acceptance*. The respondents’ acceptance of 17 climate-protection regulations was assessed based on the suggestions of Germany's Citizens’ Climate Council [10,24]. The items covered four domains: general policy statements, energy and housing, mobility, and diet and food production. Agreement with the statements ranged from “completely disagree” (1) to “fully agree” (7). A mean score was calculated across all measures (*M* = 4.8, *SD* = 1.3, *α* = 0.93).

*Political participation*. The items for this measure were taken from existing scales [[25], [26], [27], [28], [29], [30], [31], [32], [33]] and adapted for the climate topic and the German context [10]. The final scale consisted of 11 items reflecting conventional participation (e.g., signing petitions), activism (e.g., participating in demonstrations), and peer group-related participation (e.g., discussions with friends). The answers ranged from “never” (1) to “always”. The activism subscale has changed in the last two survey waves considered in this work. The subscale used to include an item about boycotting climate-damaging products (*n* = 1854); this was later replaced by two items about demonstrations (i.e., risking safety or legal consequences, *n* = 1976). While their means differed, both scales correlated at a high level (*r* = 0.81) and are therefore considered together. The differences in the items were marked in the questionnaire (https://osf.io/f2vu4). A mean score across all items was calculated (*M* = 3.1, *SD* = 1.4, 7; *α* = 0.93).

## 3 Results

### 3.1 Participants

Four survey waves were combined (August 2022–January 2023), resulting in a sample of *N* = 3830. The participants were aged between 18 and 74 years (*M* = 46.42, *SD* = 15.24). Regarding gender, 49.53% of the respondents chose male, and 50.21% selected female; 10 participants indicated nonbinary. The majority of participants (54.96%) had a university degree. Table 1 presents a detailed overview of the sample characteristics.

**Table 1:** Overview of participants’ characteristics across the four samples. Differences in sums arise from the option not to specify personal information (relevant for chronic condition, income, and migration).

Variable	*Line total*	n per survey date			
		*16.08.22*	*27.09.22*	*08.11.22*	*17.01.23*
**Age**					
18–29	687	194	159	155	179
30–49	1419	261	384	374	400
50–64	1145	286	292	281	286
65–74	579	122	156	150	151
*sum*	3830	863	991	960	1016
**Gender**					
male	1897	431	484	487	495
female	1923	430	505	468	520
nonbinary	10	2	2	5	1
*sum*	3830	863	991	960	1016
**Education**					
low	404	94	118	107	85
medium	1321	323	348	327	323
high	2105	446	525	526	608
*sum*	3830	863	991	960	1016
**Municipality size** (number of inhabitants)					
≤ 5.000	626	159	153	161	153
5.001 - 20.000	876	203	212	222	239
20.001 - 100.000	978	198	284	234	262
100.001 - 500.000	648	146	157	169	176
> 500.000	702	157	185	174	186
*sum*	3830	863	991	960	1016
**Employment status**					
employed	2684	601	692	674	717
unemployed	1146	262	299	286	299
*sum*	3830	863	991	960	1016
**Chronic condition**					
chronic condition	1349	344	328	339	338
no chronic condition	2405	509	645	599	652
*sum*	3754	853	973	938	990
**Having children**					
children	1132	285	296	281	270
no children	2698	578	695	679	746
*sum*	3830	863	991	960	1016
**Income per month**					
1250€ to below 1750€	680	174	174	174	158
1750€ to below 2250€	844	194	214	209	227
2250€ to below 3000€	834	169	224	202	239
3000€ and more	553	129	129	148	147
below 1250€	683	147	180	170	186
*sum*	3594	813	921	903	957
**Migration history**					
yes	533	108	125	155	145
no	3282	750	861	801	870
*sum*	3815	858	986	956	1015

### 3.2 Indicators of readiness to act

**Fig. 1:**
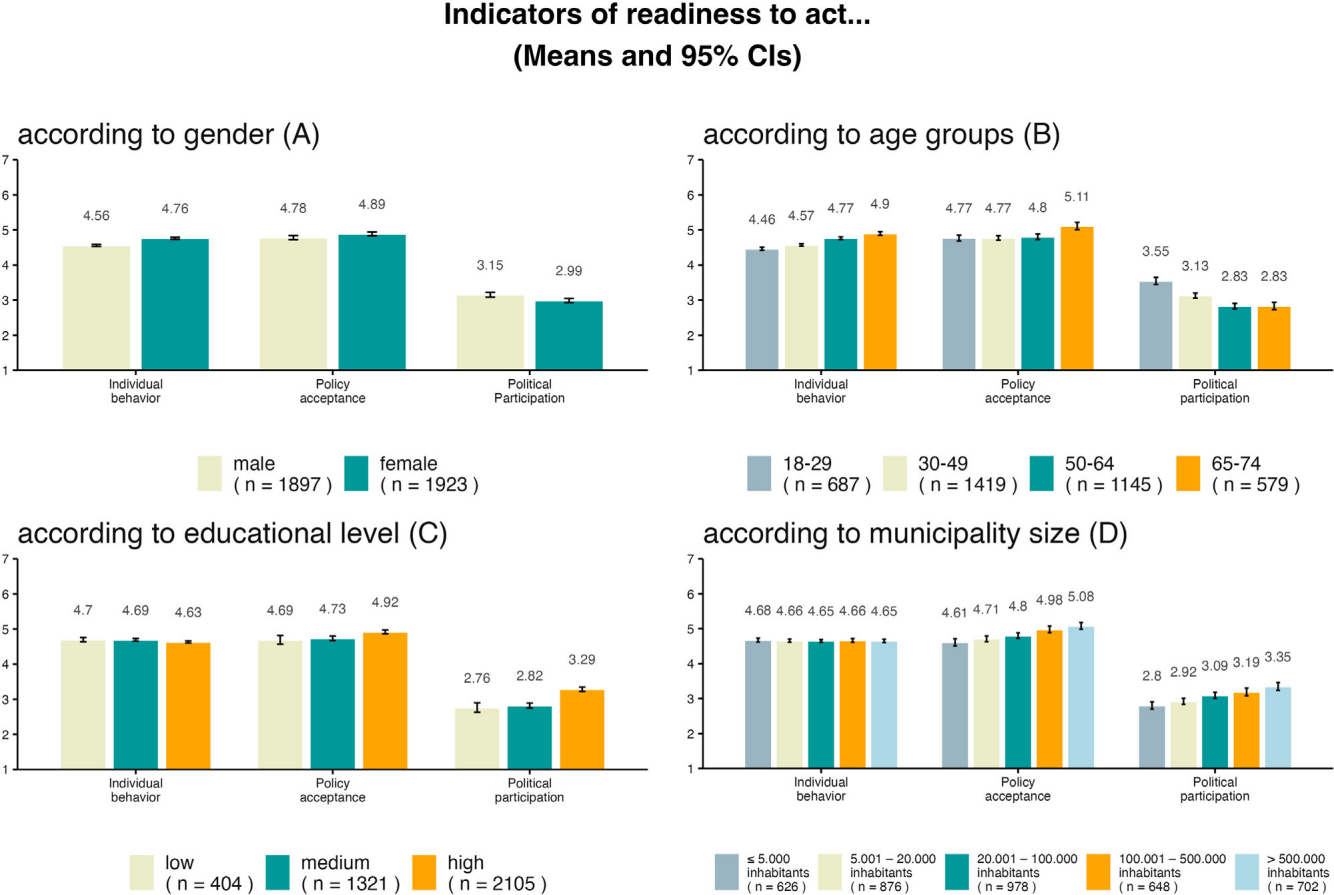
Indicators of the readiness to act against climate change as a function of gender (A), age (B), education (C), and municipality size (D). *Note.* The figure shows the effects of gender, age, education, and municipality size on the three single indicators of readiness to act: individual behavior, acceptance of policy measures, and political participation. Means and 95% CIs: *N_A_* = 3820, *N_B-__D_* = 3830.

This study aimed to explore effects of sociodemographic variables that have been found to influence people's overall readiness to act [11] on specific indicators. As gender, age, education, and municipality size produced differences in the readiness to act, we chose these variables to assess their effects on the three indicator variables. T-tests (gender, two groups only) or ANOVAs (all other variables, more than two groups) were calculated to check for significant differences. The assumption of homogeneity of variances for *t*-tests was verified using Levene's tests. In cases where Levene's tests indicated significant variance in heterogeneity, non-parametrical Welch's tests were conducted to ensure robustness. The 95% confidence intervals (CIs) in Fig. 1 allow comparison of the means between the single categories [34]. If the comparisons indicated equality, a Wilcoxon test was performed to verify these findings. The same analyses were conducted with sociodemographic factors that had not been found to be significant predictors of readiness to act [11]. These results are presented in the supplement (https://osf.io/f2vu4). Less individual climate-friendly behavior is shown by people with higher income. Less individual behavior as well as more participation is shown when a person is a parent, has a history of migration, and does not have a chronic illness. In contrasts, acceptance of policy measures showed no significant differences based upon income, parenting status, migration status and presence of a chronic illness.

Fig. 1A shows that women exhibited more climate-friendly behavior than men (*t*(3818) = −9.58, *p* < .01, *d* = −0.31) as well as more acceptance of political measures (*t*(3818) = −2.57, *p* = .01, *d* = −0.08). However, concerning political participation, men showed slightly higher values than women (*t*(3818) = 3.69, *p* < .01, *d* = 0.12). Levene tests were significant for policy acceptance (F(3818) = 14.73, *p* < .01) and political participation (F(3818) = 24.75, *p* < .01). Nonparametrical Welch's *t*-tests were therefore conducted and yielded the same results and effect size for both policy acceptance and political participation.

Age was segmented into groups as shown in Fig. 1B. Older people tended to have higher scores than the young for individual behavior (*F*(3826) = 65.02, *p* < .01, *η^2^* = 0.05) and policy acceptance (*F*(3826) = 11.36, *p* < .01, *η^2^* < 0.01); the opposite trend was observed for political participation (*F*(3826) = 45.93, *p* < .01, *η^2^* = 0.03). Regression analyses with a continuous age variable revealed the same trends.

Fig. 1C shows that higher education was related to less climate-friendly individual behavior (*F*(2, 3827) = 4.23, *p* = .015, *η^2^* = 0.002) but greater acceptance of policies (*F*(2, 3827) = 11.59, *p* < .01, *η^2^* = 0.006). Furthermore, individuals with high education showed higher levels of political participation (*F*(2, 3827) = 57.64, *p* < .01, *η^2^* = 0.03) than people with low and medium education.

Fig. 1D shows no meaningful differences for municipality size regarding individual behavior (*F*(4, 3825) = 0.161, *p* = .96). A nonparametric Wilcoxon test (TOST, two one-sided tests [35]) also indicated no between-group differences (*p_equ_* < 0.01). For the other two indicators, there were positive trends linked to increasing municipality size: People in (larger) cities were more likely to accept policies for more climate protection (*F*(4, 3825) = 16.3, *p* < .01, *η^2^* = 0.02), and they showed more frequent political participation (*F*(4, 3825) = 16.54, *p* < .01, *η^2^* = 0.02).

### 3.3 Age effects

As discussed above, the effect of age was particularly interesting. Therefore, we explored the correlations between age and the category means (i.e., means across all items on housing, on mobility, food, and other consumption). Then, correlations with the single items were inspected. For age we used the continuous variable to minimize the loss of information on available data. The variables were recoded so that higher values on the categories and items pertained to more climate-friendly behaviors, greater policy acceptance, and more frequent political participation. The correlations were considered from *r* > |.1|.

*Individual behavior.* Age was positively correlated to housing (*r* = 0.13, *p* < .01) and mobility behavior (*r* = 0.18, *p* < .01) as well as other consumption (*r* = 0.19, *p* < .01)—that is, the older people were, the more they showed climate-friendly behavior in these areas. There was no significant correlation between age and nutritional behavior (*r* = 0.03, *p* = .11). At the single-item level, there were significant correlations (*r* > |.1|, *t*(3828) > |8.38|, *p* ≤ 0.01). Older individuals were found to live in larger flats/houses (higher CO_2_ impact), they showed more climate-friendly venting behavior and tended to shower shorter than younger individuals. There were no relations of age with the isolation of flats/houses as well as room temperature. Concerning mobility, the older individuals were, the less CO_2_-impactful behavior they demonstrated by driving and flying less. Regarding nutrition, age was positive related to using groceries before they spoil and buying fewer air-transported groceries. The amount of meat, eggs, and dairy products consumed and the frequency of buying ecological groceries were unrelated to age. Yet, the older people were, the more they purchased energy-efficient and sustainable products; bought only things they needed and bought more durable items, which they used as long as possible and tended to repair rather than replace. However, the older individuals were, the less likely they purchased secondhand goods. There were negligible or no correlations between age and the choice of sustainable products, as well as selling or donating unused items to others.

*Policy acceptance*. We found the highest correlations between age and policy acceptance in the acceptance of general policies (*r* = 0.12, *p* < .01) and mobility policies (*r* = 0.13, *p* < .01), while there were no significant correlations with energy (*r* = 0.02, *p* = .18) or nutrition policies (*r* = 0.03, *p* = .08). There were significant correlations at the item level (correlations: *r* > 0.1, *t*(3828) > 6.6, *p* < .05): The older participants were, the more likely they agreed that the climate transition should be intergenerationally just and that climate-relevant actions must have direct impacts on those who cause damage. Higher age was also linked to agreeing that renewable energy should always be less costly than fossil energy. Furthermore, the older people were, the more they tended to be in favor of an expansion of public transport and the abolishment of within-country short-haul flights. There were negligible relations of age with nutrition policies, the expansion of the railway network, the provision of land for renewable-energy installations, the refurbishment of old buildings, and a climate-neutral future economy.

*Political participation.* Age was negatively correlated with conventional participation (*r* = −.19, *p* < .01), activism (*r* = −.20, *p* < .01), and peer group-related participation (*r* = −.12, *p* < .01). At the item level, there were several significant correlations (correlations: *r* > 0.1, *t*(3828) < −7.9, *p* < .01). The younger people were, the more frequently they signed petitions and took action in their communities. They tended to demonstrate and risk their safety or legal consequences more frequently than older individuals. Furthermore, the younger people were, the more often they commented on political issues online, argued against remarks opposing their opinions, and influenced other people in their political opinions and voting choices. The extent to which the respondents held discussions in their offline social environments, boycotted climate-damaging products, and voted was not linked to age.

## 4 Discussion

This study aimed to better understand the links between sociodemographic variables and the single indicators of the readiness to act against climate change. Previous work [11] found that general readiness to act in this domain differed as a function of age, gender, education level, and community size. Hence, we wished to learn whether there were differential patterns across sociodemographic variables when inspecting separate indicators of the readiness to act, namely individual behavior, policy acceptance and political participation.

In summary, more climate-friendly individual behaviors were shown by women, older participants, and those with higher levels of education, whereas community size had no discernible effect. Policy acceptance was predicted by female gender, higher age, higher education, and a larger community size. A higher frequency of climate-related political activities correlated with being male, young, highly educated, and living in a larger community.

Some of our findings challenge previous research that has associated higher age with greater CO_2_ emissions (US sample [36]) and less policy acceptance (German sample [37]). They also challenge the image of an active, pro-environmental youth. Regarding individual behavior, one explanation might be that older people's actions are based on low-emission habits (flying used to be expensive, tropical fruits were not or rarely available, etc.). Another possibility is that the young are busy with other problems that directly affect their lives (e.g., finding employment) [18]. Other scholars have suggested that current evidence about young individuals could show late effects of the COVID-19 pandemic on their lives [38]. According to some studies, while young people often identify structural reasons for climate change, they tend to focus on minimal individual-level actions, such as energy conservation, rather than participate in actions aimed at producing structural change, such as demonstrations [18,39,40]. Thus, according to Corner et al. [18], “it may be that young people are no different to older populations in many regards” (p. 530). For certain behaviors, our results are similar to those of previous research; for example, older people took shorter showers than younger individuals [20]. The conflicting findings regarding age could benefit from looking at an even younger target group—for instance, those aged 13 years and above, who might have a different awareness and understanding of climate change. The distinct results may also stem from methodological differences, such as explicitly asking about high-impact behaviors in our study, whereas other studies also include low-impact behaviors (such as switching off lights [20]). In addition, another reason for the different results may lay in potential sample distortions, particularly concerning an above-average education level in our study.

In general, the effects of the sociodemographic variables were rather small. Models such as the PACE model [10] or other models aiming to explain sustainable behaviors, policy acceptance or political participation assume that socio-cognitive factors play a crucial role in explaining individual differences in these outcomes (for an overview, see [9,10]). Still, the present study shows that the obtained knowledge on the differential effects of sociodemographics can have consequences for adapting climate communication to cater to individuals of different ages or living in municipalities of different sizes.

It is important to note some limitations regarding our study design. The study used quota-representative sampling which may lead to self-selection bias. As the sample is not a probabilistic one, generalizability of the results is limited. All measures were self-reports and may lead to estimation errors where exact answers are unknown (e.g., in the municipality size item). Furthermore, all findings reported here are correlative; thus, they cannot provide insights into potential causal relationships. It is noteworthy that the scales employed for output variables were newly developed to accurately represent real-world, high-emission scenarios [9] and this might explain some differences compared to previous research [20], where, for example, low emission behaviors such as interior lighting were also considered. It also needs to be taken into account, that the indicators of the readiness to act were not assigned specific weights relative to each other, which means that it is not possible to quantify the overall climate impact, e.g., to which degree a high individual footprint (i.e., a high CO_2_ impact) can be offset by a considerable handprint [41], such as active political participation.

However, some of the effects we found are (very) small (*η^2^* < 0.01 and *r* < 0.3). Therefore, the impacts of targeted communication may be limited in a real-world setting. Nevertheless, when scaled to an entire population and given the limited time frame regarding climate protection, any improvement in communication efficiency can be substantial. Future research could explore how to use psychological factors underlying a target group's policy acceptance, for example, e.g., by testing interventions that target specific cognitive influences or by enhancing the perceived effectiveness of measures or stressing the health risks caused by climate change [9,10]. In general, experimental studies will help to gather causal insights and longitudinal surveys will help to observe changes over time (e.g., regarding whether and if so, why individual behavior becomes more climate friendly as people age).

## 5 Conclusions

Depending on the specific focus of climate relevant behaviors, different target groups may exhibit varying potential for change. For instance, our results suggest that older people could be addressed to instigate political participation, while people in small communities seem to be important target groups to advocate for policies for more climate protection. We conclude that, overall, sociodemographic factors are relevant to health and climate communication, but as can be derived from the rather small effect sizes, the impact is rather small. Hence, future communication and campaigning strategies should leverage these insights by considering the specific action levels of their target groups in relation to the intervention focus. The finding that young people fall short regarding two aspects of readiness to act compared to older individuals (contrary to common perceptions) underscores the need to include them in climate-change communication efforts.

## Ethical clearance

The research obtained ethical clearance from the University of Erfurt's Institutional Review Board (approved June 29, 2022). The participants provided written informed consent before taking the survey.

## Declaration of generative AI and AI-assisted technologies in the writing process

During the preparation of this work, the authors used ChatGPT (version 3.5) to support translation processes and refine the use of the English language. After using the tool, the authors reviewed and edited the content as needed. They take full responsibility for the content of the publication.

## Author agreement statement

We the undersigned declare that this manuscript is original, has not been published before and is not currently being considered for publication elsewhere. We confirm that the manuscript has been read and approved by all named authors and that there are no other persons who satisfied the criteria for authorship but are not listed. We further confirm that the order of authors listed in the manuscript has been approved by all of us. We understand that the Corresponding Author is the sole contact for the Editorial process. He/she is responsible for communicating with the other authors about progress, submissions of revisions and final approval of proofs

## CRediT authorship contribution statement

**Lena Lehrer:** Writing – review & editing, Writing – original draft, Project administration, Investigation, Formal analysis, Data curation, Conceptualization. **Lennart Hellmann:** Formal analysis, Data curation. **Cornelia Betsch:** Writing – review & editing, Supervision, Methodology, Funding acquisition, Conceptualization.

## Declaration of competing interest

The authors declare that they have no known competing financial interests or personal relationships that could have appeared to influence the work reported in this paper.

## Data Availability

Details of the methods are provided online at https://osf.io/f2vu4.
